# Psychological distress and mother-child relationship: influence of life context on a population sample (BRISA) through the use of directed acyclic graphs (DAG)

**DOI:** 10.1590/1414-431X202010080

**Published:** 2020-12-07

**Authors:** M.C.V. Cavalcante, Z.C. Lamy, A.K.T.C. França, M.U.L. Pereira, A.A. Ferraro, M.A. Barbieri, F. Lamy-Filho

**Affiliations:** 1 Hospital Universitário, Universidade Federal do Maranhão, São Luís, MA, Brasil; 2 Departamento de Saúde Pública, Universidade Federal do Maranhão, São Luís, MA, Brasil; 3 Departamento de Ciências Fisiológicas, Universidade Federal do Maranhão, São Luís, MA, Brasil; 4 Departamento de Saúde Coletiva, Universidade Estadual de Campinas, Campinas, SP, Brasil; 5 Departamento de Pediatria, Faculdade de Medicina, Universidade de São Paulo, São Paulo, SP, Brasil; 6 Departamento de Puericultura e Pediatria, Faculdade de Medicina de Ribeirão Preto, Universidade de São Paulo, Ribeirão Preto, SP, Brasil; 7 Departamento de Medicina III, Universidade Federal do Maranhão, São Luís, MA, Brasil

**Keywords:** Mental suffering, Mother-infant interaction, Mother-child relations, Association, Psychosocial impact

## Abstract

This study aimed to investigate the association between maternal psychological distress and impairment in mother-child relationship in a sample from a Northeast capital city in Brazil with a low Human Development Index, using directed acyclic graphs (DAG). A total of 3,215 women were evaluated for the presence of psychological distress through the Self Reporting Questionnaire instrument and for the mother-child relationship by the first factor of Postpartum Bonding Questionnaire, considered the most appropriate in the literature. Demographic and socioeconomic variables were used to construct a theoretical model and, after this, multivariate logistic regression was performed using variables suggested by Directed Acyclic Graphs (DAG). Psychological distress was present in 22.7% of the women and 12.6% of them presented impaired mother-child relationships. After adjustment, the variable ‘maternal mental distress' remained associated with impaired mother-child relationship (RR=3.03), and among the explanatory variables only ‘primary school level' (RR=1.48) was associated as a risk factor to this outcome. The results indicated that, in this population, women with psychological distress and lower schooling are more likely to present impaired mother-child relationships.

## Introduction

A mother’s mental health is fundamental to provide an ideal environment for a child's growth in early childhood (1), since the mother constitutes a significant part in the social environment of the baby and mediates his/her experience in the outside world (2). One of the ways to assess mental health is by measuring psychological suffering, also called minor mental disorder, characterized by Coutinho (3) as a non-specific discomfort with physiological and psychological repercussions that can result in severe limitations in activities of daily life and become a disease depending on its intensity and chronicity. At first, the issues manifest in a subtle way and not necessarily lead to an immediate search for medical intervention. However, the condition can affect individuals in their relationships, behaviors, and emotions, and can be a gateway to other disorders and psychopathological manifestations (3).

Depression, anxiety, and stress are the most commonly investigated and discussed types of maternal perinatal psychological distress (4). The data reveal that it is a common and long-lasting phenomenon but subject to change (5) and it is recurrent in pregnancy (6). A substantial proportion of women who experience it during this period or in the immediate postpartum continue to show symptoms throughout the child's first years of life (7).

This type of suffering has been related to premature labor, cesarean section (8 [Bibr B09]), reduced breastfeeding (8–10), low economic status (11), unplanned pregnancy (11,12), unemployment (13), lack of social support (12), conflicting marital relationships (14), depressive symptoms (13,14), chemical dependence (15), domestic violence (12), and ambivalence towards the fetus (10).

Maternal psychological distress can also negatively affect the mother-child relationship due to difficulties of the mother in interacting (10,16) and bonding with the child (1,10), generating risk for further issues in infants who do not form a close relationship with a caregiver before the age of two years (17).

It is known that aspects related to the conditions of life, such as socioeconomic and demographic characteristics of mothers, can influence both the mother-child relationship (18) and maternal psychological distress (12). According to Ferreira and Lima (19), mothers in risky social contexts may present less responsive interaction patterns, marked by verbal negativity, little recognition and appreciation of the child's initiatives, little affective warmth, and less sensitivity to signals presented by the child.

This association between maternal psychological distress and impaired mother-child relationships is well-established in the literature (10,16,20–22); however, few studies have evaluated which maternal characteristics may interfere in this association, especially among mothers in less favorable contexts of life (19,23). Ribas and Moura (24) highlight that it is still necessary to investigate this issue in different regions and considering the family's socioeconomic context. Population-based studies controlled for different socioeconomic factors are also necessary. Research methods that identify variables for which the model should be adjusted and variables for which control is inadequate or unnecessary are recommended.

Thus, the aim of this study was to investigate the association between maternal psychological distress and impairment in mother-child relationship in a sample from a Northeast capital city in Brazil with a low Human Development Index (HDI), using directed acyclic graphs (DAG).

## Material and Methods

This study is part of the research entitled “Etiological factors of preterm birth and consequences of perinatal factors on child health: Birth cohorts in two Brazilian cities - BRISA”, carried out in Ribeirão Preto (SP) and São Luís (MA). In the current study only data from the city of São Luís were used. This city is the capital of Maranhão, a northeastern state that has the second worst HDI in Brazil (25).

A total of 3,215 mothers of children aged between 15 and 36 months living in the city of São Luís were included. They were evaluated for the mother-child relationship between April 2011 and March 2013. They were part of the BRISA birth cohort that interviewed 5,167 women immediately after delivery in the period from January to December 2010, thus corresponding to a response rate of 62.2%. To reduce the influence of loss to follow-up in the analysis, a weighting procedure was carried out considering the differences in the distribution of two variables (economic classification and gestational age) between the group of lost participants and the analyzed group.

The sample was stratified by maternity center, with a share proportional to the number of births, excluding those centers with less than 100 births per year. A systematic sampling was performed in which one woman was randomly selected for every three births. The minimum sample size at birth was set at 5000. Further information on the BRISA Cohort methodology is detailed by da Silva et al. (26).

Data on living conditions, demographic, and socioeconomic characteristics of the women collected at birth were used. Impaired mother-child relationship and maternal psychological distress were evaluated in the follow-up assessment, when the children were between 15 and 36 months old.

The explanatory variables were collected from a structured questionnaire and categorized as follows: marital status (with or without partner), number of children with whom they live (none, 1, 2-3, ≥4), schooling (up to middle school; incomplete high school to college degree), paid activity (yes or no), family income (assessed in minimum salaries and categorized as <3, ≥3 and <5, ≥5) and type of childbirth care (financed or not by SUS - Brazilian Unified Health System).

The exposure variable was maternal psychological distress evaluated through the Brazilian version of the Self Reporting Questionnaire (SRQ-20). This tool measures the existence of mental distress or minor psychiatric disorders, and it was validated for the Brazilian population by Mari and Willians (27). It is considered to have rapid and easy application, to be reliable, and to have high discriminating power. Moreover, it is a valuable tool for population-based studies aimed at the identification of non-psychotic mental disorder cases (28). The version of the instrument used has 20 yes-or-no questions that provide a score, with which it is possible to identify the presence of psychological distress and a likely case of common mental disorder. The total score is calculated by adding affirmative answers, and has a minimum of zero and a maximum of 20 (29). Respondents with seven or more positive responses are considered to have psychological distress (27).

Impaired mother-child relationship, the outcome variable, was evaluated through the Portuguese version of the Postpartum Bonding Questionnaire (PBQ) (30). It is used for the diagnosis of disorders in this relationship (31), has adequate levels of sensitivity (32), reliability, and validity, and can be used in clinical and research contexts (30).

The PBQ is a self-reported instrument with 25 items rated in a Likert-type scale as “always”, “usually”, “frequently”, “sometimes”, “rarely”, and “never”, ranging from zero to five (31), in which the highest values indicate relationship difficulties and lower values, favorable situations. Some items had the rating direction in reverse order (items 2, 3, 5, 6, 7, 10, 12, 13, 14, 15, 17, 18, 19, 20, 21, 23, and 24) (33).

For analysis, only the first factor of the PBQ was used, which measures the characteristics of the mother-child relationship (33), considered the most adequate, especially to evaluate mothers in the general population (22). The first factor, which contains twelve items, is called “impaired bond with the baby” and assesses the level of positive feelings and proximity to the baby. It varies from 0 to 60 points, with values greater than or equal to 12 classified as pathological (30).

In the descriptive analysis, the categorical variables are reported as frequencies and percentages. The reliability evaluation of the PBQ revealed adequate internal consistency verified with Cronbach's alpha coefficient of 0.8.

**Figure 1:**
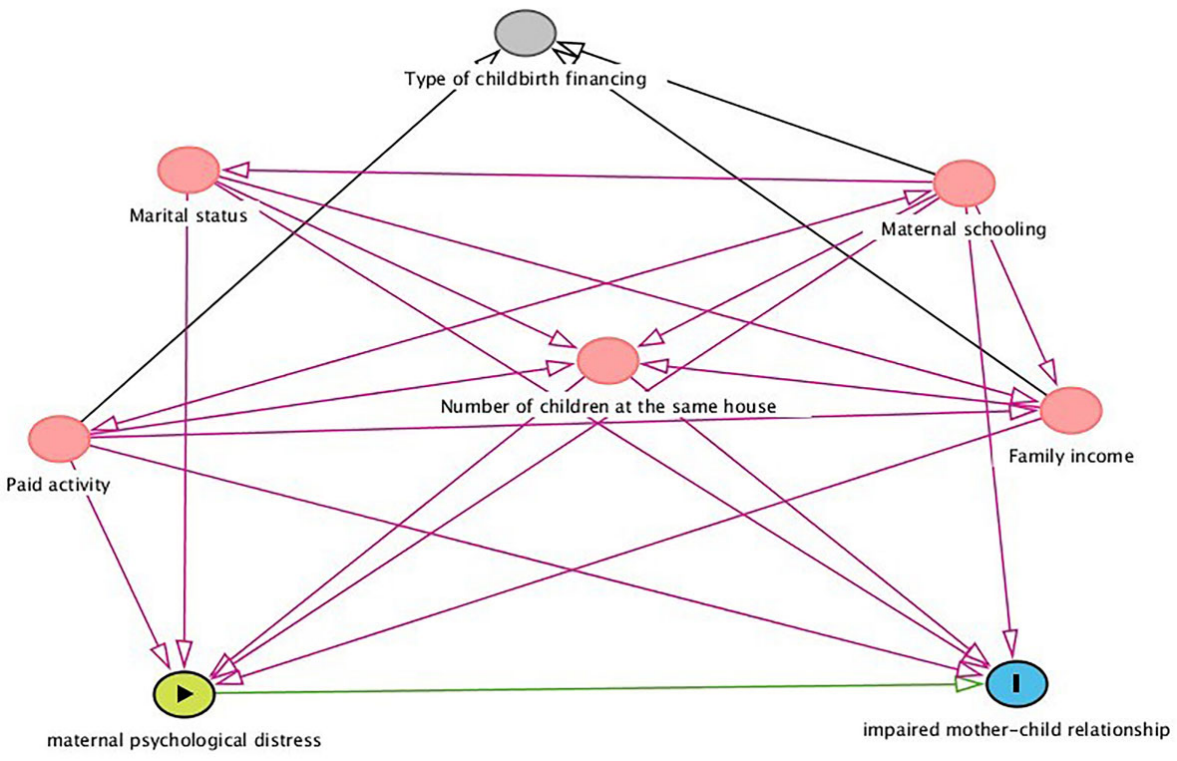
Directed acyclic graph of the association between psychological distress and mother-child relationship. Green: exposure; blue: outcome; pink: ancestor of exposure and outcome; gray: other variable.

An explanatory theoretical model was built based on demographic and socioeconomic variables established in the literature that could have some influence on the association between psychological distress and mother-child relationship, which was subsequently analyzed using DAG (Figure 1). In this model, the following variables were included: schooling, paid activity, family income, number of children living with the mother, marital status, and type of childbirth financing. The interrelations between these variables are represented in Figure 1. The variables were assumed to be related to the exposure variable and the outcome variable, being predecessors and are represented in pink color. Only the variable ‘type of childbirth financing' was considered to have no direct relationship with the outcome or exposure, and is shown in gray.

Shrier and Platt (34) report that DAG helps the identification of variables that should be considered in the evaluation of the effect of exposure on the outcome, and allows the recognition of those for which control is inappropriate or unnecessary.

By using this model and the program DAGitty 3.0 (public domain) for the evaluation of the cumulative effect, the following variables were used in the adjustment: education, paid activity, family income, number of children living with the mother, and marital status. Then, logistic regression analysis was performed, and the variables with a P-value <0.05 were considered significant. The relative risk (RR) and their respective 95% confidence intervals were also estimated. The regression analysis was performed in the Stata^®^ 12.0 statistical software (StataCorp, USA).

BRISA was approved by the Research Ethics Committee of the University Hospital of UFMA (Clinical Opinion No. 223/2009) and Hospital das Clinicas of the Medical School of Ribeirão Preto (Official Letter 4116/2008) in compliance with the criteria of Resolution 196/96 of the National Health Council and its amendments, which was effective at the time.

## Results

A total of 3,215 mothers of children aged 15 to 36 months were evaluated, corresponding to a response rate of 62.2% of the 5,167 women interviewed at the birth of their children. The losses were caused by difficulty in locating the mother and mother refusal.

The sample consisted predominantly of women aged between 20 and 34 years (73.7%), who had a partner (80.6%), and had schooling ranging from unfinished high school to college degree (77.2%). Most of them did not have paid work (65.6%), had a family income of less than three minimum wages (66.6%), and had given birth in hospitals of the Unified Health System (SUS) (85.1%) (Table 1).

**Table 1:** Demographic and socioeconomic characteristics of the mothers from the BRISA Study, São Luís, 2013.

Variables	n (%)
Age (in years)	
<15	22 (0.7)
15-19	550 (17.1)
20-34	2372 (73.8)
≥35	271 (8.4)
Marital status	
With partner	2598 (80.6)
Without partner	617 (19.4)
Number of children living with the mother	
None	1601 (49.7)
1	1032 (31.9)
2-3	506 (16.0)
≥4	76 (2.4)
Maternal schooling^*^	
≤9 years	731 (23.4)
>9 years	2472 (76.6)
Paid activity	
Yes	1101 (34.4)
No	2114 (65.6)
Family income (assessed in minimum wages)^*^	
<3	1790 (66.6)
≥3 and <5	439 (16.0)
≥5	440 (17.4)
Type of childbirth financing	
SUS**	2756 (85.1)
Not by SUS	459 (14.9)

*n<3215. **SUS: Brazilian Unified Health System.

Psychological distress was observed in 22.7% of the assessed women, of which 12.6% presented impaired mother-child relationship (data not shown in the table).

When structuring the theoretical model through DAG, a potential influence of all the variables included was verified, except for the variable ‘type of childbirth financing', which was not included in the group of variables used for adjustment.

An association between maternal psychological distress and impaired mother-child relationship was found in this study (RR=3.03, 95%CI: 2.46-3.74). Concerning the socioeconomic and demographic factors investigated in the study, only lower maternal schooling was associated with impaired mother-child relationship, who had 48% higher risk than those with higher educational level (Table 2).

**Table 2:** Adjusted logistic regression analysis of the association between maternal psychological distress and impaired mother-child relationship, from the BRISA Study, São Luís, 2010.

Variables^*^	RR (95%CI)	P-value
Impaired mother-child relationship		
Yes	3.03 (2.46-3.74)	<0.001
No	1	
Marital status		
With partner	1	
Without partner	1.18 (0.92-1.51)	0.185
Number of children living with the mother		
None	1	
1	0.96 (0.76-1.22)	0.759
2-3	0.96 (0.73-1.26)	0.727
≥4	0.85 (0.46-1.46)	0.463
Maternal schooling		
≤9 years	1.48 (1.19-1.85)	0.001
>9 years	1	
Paid activity		
Yes	1	0.973
No	0.99 (0.79-1.26)	
Family income (assessed in minimum wages)		
<3	0.85 (0.55-1.31)	0.462
≥3 and <5	1.16 (0.82-1.65)	0.406
≥5	1	

*Adjustment variables suggested by the directed acyclic graphs. RR: relative risk; CI: confidence interval. Significant P-value <0.05.

## Discussion

After adjustments for the variables suggested by DAGitty, the total effect of the association between maternal psychological distress and impaired mother-child relationship remained statistically significant.

The results of this study confirmed the influence of psychological distress on impaired mother-child relationship, previously established in the literature. Women with this type of suffering had a 3.03 times greater risk for impaired mother-child relationship over the second and third years of the child's life compared to those who did not present psychological distress.

Although the birth of a child should be a moment of joy, it also brings sudden changes in the mother's life (35) and can become a stressful event (36), possibly generating psychological problems (35). Greinert et al. (10) reported that mothers with psychological distress in the postpartum period may have ambivalent feelings towards the baby and difficulties bonding with their children. This can interfere in the establishment of an appropriate emotional bond between the dyad.

Corroborating these findings, Horwitz et al. (37) reports that a substantial proportion of women experiencing psychological distress in pregnancy or over the postpartum period continues to experience the symptoms during the first years of life of their children, and may present difficulties in bonding (16).

These mothers should receive additional support to reduce the risk of delay in the overall development of their children, since the inconsistent bond may also have a direct influence on emotional and behavioral damage throughout childhood and adult life (20).

The literature indicates that less favorable contexts of life have a negative repercussion on the mother-child relationship (38). Nevertheless, among the variables that were part of the model, income, paid activity, marital status, and number of children living with the mother were not associated with impaired mother-child relationship.

Ferreira and Lima (19) evaluated 30 dyads regarding the relationship between psychosocial risk and quality of interaction between mothers and children of preschool age in Portugal. The authors identified a worse interaction among mothers with low educational level, with precarious employment or unemployed, and in families with low economic level. Such conditions could negatively influence the quality of maternal interaction because they are a source of family stress and can increase the difficulty of these mothers to recognize and respond to the child's signals (19).

In this same context, Stack et al. (23) evaluated the emotional availability of 4,109 mothers with psychosocial risk in a cohort in Canada. They concluded that financial security, educational level, affective warmth, and sensitivity of parents are protective factors for the mother-child relationship and predict positive results for the children. According to these authors, positive, stable, and stimulating domestic environments allow the relationship to be established in a healthier way.

Both studies pointed to the influence of lower educational level, low income, and the absence of paid activity in impaired mother-child relationship, despite the methodological difference between them.

In the current study, only the lowest educational level was a risk factor to an impaired relationship. This variable is associated with most infantile disorders and has been shown to be important for the mother-child relationship. Differently from the Canadian study, we did not find an association with income and paid activity, and the difference between studies could be the different social indicators of the countries. Of note, Maranhão has the second worst HDI among the Brazilian states (25), and Brazil ranks 38th in cognitive capacity and school success among 40 countries (39).

According to the findings of Serradas et al. (38), women with higher educational level have better involvement with the child. Parental education can offer benefits beyond material resources made possible due to higher income, as they would have greater access to information that may influence parental care and the developmental needs of their children (40).

The use of a questionnaire for the evaluation of the mother-child relationship was considered a limitation in this study, as the “gold standard” would be the direct observation of the dyad. Despite this, the PBQ is a validated and widely used instrument for such an assessment. Another limitation was the lack of data on type of housing and leisure of the evaluated women, which could make the characterization of the living conditions more complete. Additionally, there were losses to follow-up, which are common in cohort studies, but the bias was minimized by the use of weighting in data analysis.

On the other hand, the strengths of the study include the cohort study design performed with a large enough sample to establish the reported associations. Furthermore, the use of DAG allowed the adequate adjustment of confounding variables in the assessment of the effect of psychological distress on the mother-child relationship.

The results of this study indicated that, in the evaluated population, women with psychological distress and lower educational level are more likely to present impaired mother-child relationships.

Assessing the risk of psychological distress and other factors that negatively affect the mother-child relationship is of extreme importance for the development of public policies and actions to prevent and mitigate relationship problems.
